# Supplementary material for: Cellulose Aerogel Derived Hierarchical Porous Carbon for Enhancing Flavin-Based Interfacial Electron Transfer in Microbial Fuel Cells
Source: Polymers (Basel). 2020 Mar 17;12(3):664. doi: 10.3390/polym12030664 (PMC7183089; doi:10.3390/polym12030664)
Supplement: Supplementary file 1 [file polymers-12-00664-s001.pdf]

Supplementary Materials

Cellulose Aerogel Derived Hierarchical Porous Carbon for Enhancing Flavin-Based Interfacial Electron Transfer in Microbial Fuel Cells

Deng Wang [1, 2] Ying Wang [1, 2], Jing Yang [1, 2], Xiu He [1, 2], Rui-Jie Wang [1, 2], Zhi-Song Lu[1, 2, *] and Yan Qiao [1,2,*]

[1] School of Materials and Energy, Southwest University, Chongqing 400715, China; wonderfulstarlight@163.com (D.W.); wonderying819@163.com (W.Y.); jyang_0921@163.com (J.Y.); hexiuwz@163.com (X.H.); jerrywangww@163.com (R.J.W.)

[2] Chongqing Key Laboratory for Advanced Materials & Technologies of Clean Energies, Southwest University, Chongqing 400715, China

[*] Correspondence: yanqiao@swu.edu.cn (Y.Q.), zslu@swu.edu.cn (Z.S.L.);

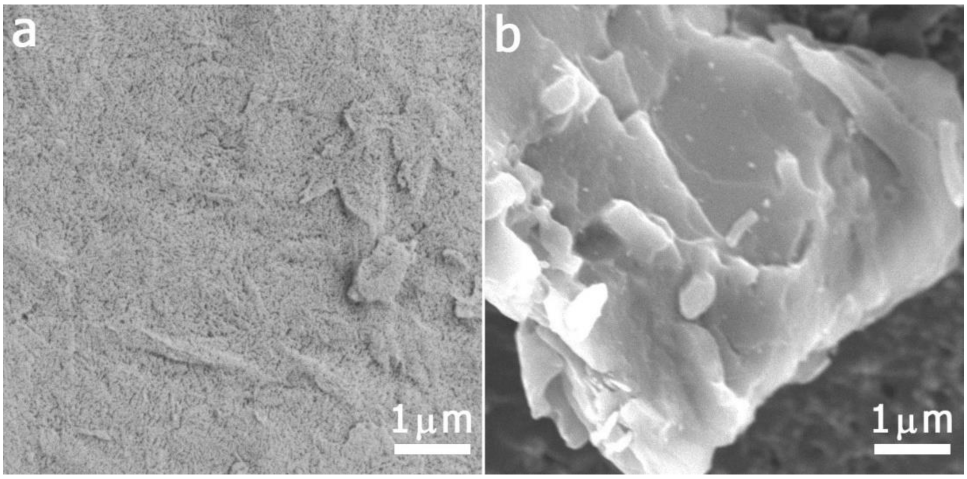

Figure S1. SEM images of cellulose powder before (**a**) and after (**b**) pyrolysis.
